# Changes in use of emergency contraceptive pills in the United States from 2008 to 2015

**DOI:** 10.1016/j.conx.2021.100065

**Published:** 2021-05-10

**Authors:** Rubina Hussain, Megan L. Kavanaugh

**Affiliations:** Guttmacher Institute, New York, NY, United States

**Keywords:** Contraceptive use, Emergency contraception, National Survey of Family Growth, Over-the-counter (OTC) access

## Abstract

**Objectives:**

To describe changes in use and receipt of emergency contraceptive (EC) pills among women in the United States during a period of key EC policy changes, from 2008 to 2015.

**Study design:**

Using data from the 2006 to 2010 and 2013 to 2017 National Surveys of Family Growth, we present changes in the percent of women who ever used EC between 2008 and 2015 by select sociodemographic and sexual and reproductive health characteristics, and we examine multivariable relationships of these characteristics with EC ever use in 2015. We also examine changes in repeat EC use, receipt of EC counseling, reasons for EC use and source of EC between the time periods.

**Results:**

Among sexually experienced women ages 15 to 44, EC ever use increased from 11% in 2008 to 23% in 2015 overall and among nearly all groups of women. In 2015, age 20 to 29, non-Hispanic other or Hispanic race, at least a high school education, working part-time, income at least 100% of the federal poverty level, ever having been married, and having received EC counseling in the prior year all represent characteristics associated with higher odds of having ever used EC. In 2015, a smaller share of women last obtained EC with a prescription or at a health facility than in 2008.

**Conclusions:**

Increases in EC use occurred as access to EC was broadened through regulatory changes that moved some forms of EC from behind-the-counter to fully over-the-counter between 2008 and 2015.

**Implications:**

Over-the-counter provision of many forms of EC pills may have increased access and introduced more flexibility in how EC is obtained, but these changes may have come with tradeoffs, both in the form of cost barriers and decreased opportunities for clinicians to discuss EC with their patients. Despite improved access to contraception more broadly through the Affordable Care Act, EC remains a necessary component of the overall contraceptive method mix, and clinicians can play a key role in discussing EC as one option among many during contraceptive counseling sessions.

## 1 Introduction

An individual's ability to have agency over their fertility preferences is dependent on their access to comprehensive reproductive and sexual health care. Within the broad mix of contraceptive methods available, emergency contraception (EC) is a unique method because it allows for postcoital pregnancy prevention when other forms of contraception may not have been used correctly or at all. EC can be especially relevant for the 8.4% of women in the United States who report having ever experienced reproductive coercion [1] and the 17.6% who report having experienced rape or attempted rape in their lifetime [2]. Both dedicated EC pills—including ella, Plan B One-Step, and generics of Plan B One-Step—and intrauterine devices (IUDs) used as postcoital contraception are included within the broader grouping of emergency contraceptive strategies.

Although dedicated EC pills first became available by prescription in the United States in 1999, the 2 dose regimen of levonorgestrel EC pills was not approved for over-the-counter (OTC) sales until 2006, when individuals aged 18 years or older could purchase EC at a pharmacy [3]. As of 2006 to 2010, the last time period for which national level data on emergency contraceptive use were published, approximately one out of every nine sexually active reproductive aged women in the United States reported having ever used EC [4].

Since that report, several events may have influenced individuals’ access to, and use of, EC. The age limit for OTC purchase of EC was lowered to 17 in 2009, but maintaining separate access strategies by age kept EC behind the counter and continued to create barriers for users of all ages [5,6]. In 2010, the FDA approved ulipristal acetate, a new and more effective form of EC [7,8], under the brand name ella for prescription-only status [9]. A one-pill regimen of levonorgestrel EC (Plan B One-Step) was approved for OTC sales for all ages in 2013, with generics of this regimen approved for OTC sales in 2014, offering the potential for unrestricted access to EC [6]. More broadly, implementation of the Affordable Care Act in 2014 increased access to the full range of contraceptive methods, including emergency contraception, by eliminating cost sharing for individuals covered by health insurance through the federal exchanges [10].

Given these shifts, this analysis documents changes in ever use of emergency contraception pills between 2008 and 2015 and identifies characteristics associated with use in the more recent time period.

## 2 Materials and methods

Our analyses draw on data from the 2006 to 2010 and 2013 to 2017 National Survey of Family Growth (NSFG) female respondent questionnaires,^1^ which include representative samples of 12,279 and 10,590 civilian, noninstitutionalized women in the United States aged 15 to 44,^2^ respectively. The questionnaires were administered in-person and the interviews were voluntary and confidential. We present results at the midpoint of each time period (2008 and 2015) as a reference year to simplify interpretation of changes between these time periods, and we used weights provided by the NSFG so that the data are nationally representative. More detailed information on the surveys are available on the NSFG website [11].

Our analytic sample includes all female respondents who indicated that they had ever had sex with a man, 85% to 86% of each cycle's sample. Our analysis examined several relevant variables in the NSFG, including ever having used EC, number of times having used EC, where EC was last obtained, whether last EC obtained was with or without a prescription, reasons for EC use (multiple responses allowed) and whether EC had been discussed during the respondent's last gynecological visit in the past year (EC counseling).^3^ The NSFG question on ever use of EC does not specifically distinguish between the types of EC pills used nor does it name IUDs as an option to consider in the EC options suggested^4^; thus EC use documented in this study captures only grouped EC pill use and not use of IUDs as EC [12].

We examined changes in EC pill use between 2008 and 2015, overall and by several demographic and sexual and reproductive health characteristics including age, race and ethnicity, education, work status, federal poverty level, marital status, religion, number of pregnancies, number of births, number of lifetime male partners, ever use of select methods of contraception, recent gynecological care and recent receipt of EC counseling. We also examined changes in several other key EC metrics (use of EC more than once, receipt of EC counseling in the past year, reasons for EC use and where and how EC was last obtained) between the 2 time points among all EC users and, for those metrics for which only potential to use EC was relevant, all sexually experienced women ages 15 to 44. We used simple logistic regression to examine bivariate relationships between demographic and sexual and reproductive health characteristics and ever use of EC in the most recent time period (2015). We then entered all demographic and sexual and reproductive health variables that were significant at the *p* < 0.05 level into a multivariable logistic regression model with ever use of EC as the dependent variable. Using backward stepwise regression, we removed any independent variables that were not significant at the *p* < 0.1 level. The final regression model included age, race/ethnicity, education, working status, poverty level, marital status, religion, number of pregnancies, number of male partners, ever use of condoms, ever use of short term hormonal birth control, and recent EC counseling. Adjusted odds ratios and 95% confidence intervals are shown for each independent variable.

All analyses were conducted using the “svy” command prefix within Stata 15.1 to account for the NSFG's use of a multistage probability sample. Our organization's institutional review board (Department of Health and Human Services identifier IRB00002197) determined that this analysis was exempt.

## 3 Results

### 3.1 Sample characteristics

Of the 12,279 women ages 15 to 44 in the 2006 to 2010 NSFG and the 10,590 women in the 2013 to 2017 NSFG, there were 10,605 and 9048 women, respectively, who reported ever having sex and were therefore considered to be potential users of EC. Changes in the distribution of sociodemographic and reproductive and sexual health characteristics of all potential and actual EC users ages 15 to 44 between 2008 and 2015 are shown in Table 1.

**Table 1:** Characteristics of sexually experienced US women ages 15 to 44, overall and who ever used emergency contraception, and *p* values from logistic regression testing significant differences between 2008 (*N* = 10,605) and 2015 (*N* = 9048), National Survey of Family Growth

Characteristic	Among all sexually experienced women		Among women who had ever used EC^a^		
	2008	2015	2008	2015	*p* value
	%	%	%	%	
All	100	100	10.8	23.1	<0.001
Age					
15–19	8.5	7.2	13.9	20.5	0.007
20–24	16.7	16.9	23.2	35.0	<0.001
25–29	18.9	19.1	15.5	35.8	<0.001
30+	56.0	56.8	5.0	15.7	<0.001
Race and ethnicity					
Non-Hispanic white	60.7	55.6	11.1	21.9	<0.001
Non-Hispanic Black	13.8	13.9	7.9	20.8	<0.001
Non-Hispanic other	8.5	10.2	12.0	26.4	<0.001
Hispanic	17.1	20.3	11.4	26.5	<0.001
Education					
Some high school or less	18.9	12.6	7.9	13.2	<0.001
High school graduate/some college	54.4	56.0	11.2	23.9	<0.001
College graduate or higher	26.7	31.4	11.9	25.7	<0.001
Work status					
Not working	30.0	28.3	9.7	18.9	<0.001
Working part-time	24.0	24.6	12.8	27.3	<0.001
Working full-time	46.0	47.1	10.4	23.5	<0.001
% offederalpoverty level					
<100	21.7	25.1	9.5	18.4	<0.001
100–499	70.2	56.6	10.7	23.7	<0.001
500+	8.2	18.3	14.4	27.9	<0.001
Marital status					
Never-married	47.9	43.4	5.6	14.8	<0.001
Ever married	52.1	56.6	15.5	29.5	<0.001
Religion					
None	18.6	23.4	16.7	30.6	<0.001
Catholic	24.8	20.8	11.0	21.4	<0.001
Protestant	48.2	48.1	8.5	20.2	<0.001
Other	8.4	7.7	10.0	23.3	<0.001
Number of pregnancies					
0	37.9	30.3	17.2	32.6	<0.001
1–4	54.3	62.7	8.4	19.5	<0.001
5+	7.9	7.0	6.9	14.9	<0.001
Number of lifetime male partners					
1	22.9	20.8	5.0	10.5	<0.001
2+	77.1	79.2	12.5	26.4	<0.001
Number of births					
0	35.8	38.0	18.1	34.7	<0.001
1–2	42.9	42.5	8.1	18.9	<0.001
3+	21.3	19.6	3.9	10.1	<0.001
Ever used a condom					
No	6.6	5.4	2.7	2.9	0.831
Yes	93.4	94.6	11.3	24.3	<0.001
Ever used short-acting hormonal methods^b^					
No	13.8	15.1	6.3	16.9	<0.001
Yes	86.3	85.0	11.6	24.4	<0.001
Ever used long-acting reversible methods^c^					
No	90.6	78.3	10.7	22.8	<0.001
Yes	9.4	21.7	11.4	24.4	<0.001
Saw gynecologist in past year forpelvic exam					
No	29.4	34.7	8.3	21.1	<0.001
Yes	70.6	65.3	11.8	24.2	<0.001

EC, emergency contraception.

a Among women who have ever had sex.

b Short-acting hormonal methods include contraceptive pills, patches, rings, and injections.

c Long-acting reversible methods include intrauterine devices (IUDs) and implants.

### 3.2 Changes in use and receipt of EC between 2008 and 2015

Ever use of EC more than doubled, from 11% in 2008 to 23% in 2015. Moreover, ever use of EC significantly increased among nearly all demographic and sexual and reproductive health groupings of women. The largest increase was among 30- to 44-year-old women (5%–16%). Having ever used EC more than doubled among a number of groups: non-Hispanic Black women, never-married women, women who had three or more births, women who had never used short term hormonal contraceptive methods (pill, injection, ring or patch) and women who had not received a recent pelvic exam. Ever use of EC remained low among women who had never used a condom—3% during both time periods.

In 2015, 10% of sexually experienced women reported using EC more than once—an increase from 4% in 2008 (Table 2). Among ever EC users, repeat use stayed relatively steady at 41% in 2008 and 45% in 2015.

**Table 2:** Changes in key emergency contraception (EC)-related metrics among ever EC users ages 15 to 44,^a^ and *p* values from logistic regression testing significant differences in these metrics between 2008 and 2015, National Survey of Family Growth

EC metric	2008	2015	*p* value
	%	%	
Use of EC more than once			
Among all sexually experienced women	4.4	10.4	<0.001
Among ever EC users	41.1	45.2	0.155
Receipt of EC counseling in year prior to survey			
Among all sexually experienced women	3.5	3.2	0.393
Among ever EC users	15.5	6.9	<0.001
Reasons for EC use			
Worried birth control would not work	45.0	41.3	0.179
Did not use birth control that time	48.8	50.2	0.596
Some other reason	12.1	15.2	0.062
Procurement of last EC			
With a prescription^b^	30.8	18.4	<0.001
At a health care facility/clinic^c^	67.4	40.2	<0.001

EC, emergency contraception.

a Unless otherwise noted; we present the first two EC metrics for both all sexually experienced US women and ever EC users.

b Only asked in years 3 and 4 of 2006 to 2010 NSFG cycle, *n* = 743.

c Only asked in years 2, 3, and 4 of 2006 to 2010 NSFG cycle, *n* = 1053.

Receipt of counseling for EC in the past year was low in both years (3%–4%). Additionally, among ever EC users, the proportion who reported having received EC counseling in the past year decreased significantly, from 16% in 2008 to 7% in 2015.

Reasons for EC use were similar in both years. In both years, a slightly higher proportion indicated they had not used birth control (49%–50%), compared to being worried that their birth control would not work (41%–45%).

Where and how women obtained EC changed significantly between the 2 periods. In 2008, 31% had a prescription the last time they used EC compared to 18% in 2015. In supplemental analyses examining this outcome by age, women across all age groups mirrored this pattern with one notable exception; young women aged 15 to 19 reported similar levels between 2008 (15%) and 2015 (13%) (not shown). The proportion of women who received EC at a health facility or clinic significantly decreased from 66% in 2008 to 39% in 2015.

### 3.3 Ever use of EC in 2015 by user characteristics

In multivariable analysis, after controlling for other user characteristics, women aged 20 to 29 had higher odds of ever using EC compared to women 15 to 19 (Table 3). Hispanic and ever-married women had higher odds of ever having used EC compared to their non-Hispanic white and never married counterparts. College graduates were more likely to have ever used EC compared to women who did not complete high school (adjusted odds ratio [aOR] = 2.4). Women with incomes above 100% of the federal poverty level were more likely to have used EC compared to women below this threshold, and the likelihood of ever use increased as wealth increased.

**Table 3:** Adjusted odds ratio and 95% confidence intervals from logistic regression analysis assessing select characteristics and women's likelihood of ever having used emergency contraception (EC),^a^ National Survey of Family Growth

Characteristic	Ever used EC		
	Adjusted odds ratio (aOR) and 95% CIs		
Age			
15–19 (ref)	1.00		
20–24	1.51	1.08	2.12
25–29	1.87	1.26	2.76
30+	0.66	0.44	0.99
Race and ethnicity			
Non-Hispanic white (ref)	1.00		
Non-Hispanic Black	0.97	0.76	1.25
Non-Hispanic other	1.51	1.12	2.03
Hispanic	1.99	1.58	2.50
Education			
Some high school or less (ref)	1.00		
High school graduate/some college	1.76	1.31	2.35
College graduate or higher	2.35	1.65	3.34
Work status			
Not working (ref)	1.00		
Working part-time	1.41	1.13	1.75
Working full-time	1.08	0.90	1.30
% offederalpoverty level			
<100 (ref)	1.00		
100–499	1.38	1.13	1.68
500+	1.81	1.40	2.34
Marital status			
Never-married (ref)	1.00		
Ever married	1.78	1.42	2.24
Religion			
None (ref)	1.00		
Catholic	0.69	0.54	0.87
Protestant	0.73	0.60	0.89
Other	1.08	0.77	1.51
Number of pregnancies			
0 (ref)	1.00		
1–4	0.77	0.63	0.95
5+	0.78	0.55	1.12
Number of lifetime male partners			
1 (ref)	1.00		
2+	2.85	2.12	3.84
Ever used a condom			
No (ref)	1.00		
Yes	5.50	2.41	12.54
Ever used short-acting hormonal methods^b^			
No (ref)	1.00		
Yes	1.55	1.25	1.92
Received EC counseling in the past year			
No (ref)	1.00		
Yes	2.75	1.82	4.17

EC, emergency contraception; CI, confidence intervals.

Note: All variables presented in the table were included in the multivariable logistic regression model.

a Among women who have ever had sex.

b Short-acting hormonal methods include contraceptive pills, patches, rings and injections.

With regards to sexual and reproductive health characteristics, women who had had at least 2 lifetime male partners were much more likely to have ever used EC compared to women who only had one lifetime male partner (aOR of 2.85). Women who had ever used a condom (aOR = 5.5) and women who had received recent counseling for EC (aOR = 2.8) were more likely to have ever used EC compared to women who had never used a condom and those who had not received EC counseling in the past year, respectively.

## 4 Discussion

Ever use of EC more than doubled from 2008 to 2015, with almost one-fourth of all sexually experienced women aged of 15 to 44 having ever used EC in the more recent time period. Increases in EC use, which were experienced across all population groups, likely reflect increased access during the time period and, potentially, the introduction of new EC pill options.

Many EC pills moved from behind-the-counter to fully OTC for all ages by the latter time period. In turn, prescriptions for EC and, thus, interactions with a clinician about EC became limited, as reflected in the drop in EC users both having obtained their last EC through a prescription and at a health care facility. These regulatory shifts in access have likely reduced time to procure EC and have potentially lessened stigma and embarrassment associated with obtaining it [13]. At the same time, both the increased odds of EC use among those who had received EC counseling from a healthcare provider and the decrease in this counseling occurring between the two time periods indicate that potential EC users may be missing a key opportunity to discuss important differences among EC options—especially the greater effectiveness of both ella and IUDs.

Individuals obtaining EC by prescription in 2015 may reflect both users of ella, which remains available only through prescription, as well as younger users of EC ages 15 to 19. These younger women, who accessed EC through prescription at the same levels in both time periods (13%–15%), may experience increased barriers to OTC EC [14]. One study conducted in 4 US states found that EC was fully available to adolescents in only 28% of pharmacies and others studies found that barriers to access persisted among adolescents after policy changes to improve access went into effect [15,16].

Higher income levels are associated with higher rates of EC use, indicating that cost may be a key barrier to accessing EC, especially given higher costs associated with OTC EC pills than those accessed through a prescription [15,17,18]. As the contraceptive coverage guarantee under the Affordable Care Act may have helped alleviate cost burden for covered individuals accessing EC through prescription, it simultaneously may have increased the cost burden for individuals accessing EC OTC given OTC methods being excluded from the contraceptive coverage guarantee [19].

Women use EC for a variety of reasons, including as back up pregnancy prevention when a primary method fails or as a sole pregnancy prevention strategy, and the percentages of women reporting these reasons has remained steady over time. Notably, women who had ever used condoms and those who had ever used short-acting hormonal methods reported higher levels of EC use than those who reported having not used these methods. These findings underscore the importance of EC as just one tool among many within the broader method mix available for pregnancy prevention options.

## 5 Limitations

This study has several limitations. Given the design of the NSFG, some population groups in the United States may be underrepresented or not represented at all, including those with English- and Spanish-language barriers, those who identify as transgender, and institutionalized individuals. The NSFG data are cross sectional and therefore temporal relationships cannot be established among the variables measured. Ever use of EC is a lifetime measure whereas many of the independent variables included in the analyses were assessed at the time of the survey. The study examines changes in EC use between two 4-year periods, with 2008 and 2015 being the midpoints of each period. Some key changes in EC policies occurred within these time periods, introducing uncertainty as to the extent to which data from the full time period reflects potential impacts. The NSFG does not distinguish between EC pills used, acknowledging that only some types (levonorgestrel options like Plan B One-Step) became available OTC during the study time frame while others (ella) remained available only by prescription. Without having a clear understanding of how much of EC use can be attributed to the different types of EC pills, it is difficult to determine how much of a role the shift in status played for users and access. Lastly, while IUDs are also considered a method of EC, the NSFG only asked about use of EC pills and may underestimate true levels of EC use in the United States.

## 6 Conclusions

OTC provision of many forms of EC pills may have increased access and introduced more flexibility in how EC is obtained [20], [21], [22], but these changes may have come with tradeoffs, both in the form of cost barriers and decreased opportunities for clinicians to discuss EC with their patients. Despite improved access to contraception more broadly through the Affordable Care Act, EC remains a necessary component of the overall contraceptive method mix, and clinicians can play a key role in discussing EC as one option among many during contraceptive counseling sessions. Continuing to work to eliminate barriers to access, as well as working to broaden EC options available—including IUDs—is essential to supporting individuals in realizing full reproductive autonomy.

## Appendix Supplementary materials

Image, application 1

## Footnotes

1 All NSFG respondents self-report their current gender at the time of interview, which determines the questionnaire they are routed into.

2 The 2015 to 2017 NFSG includes women aged 45 to 49 who were removed for this analysis.

3 The NSFG began asking where EC was last obtained and whether it was obtained with a prescription in years 2 and 3 of the 2006 to 2010 cycle, respectively.

4 The NSFG asks respondents *“(Have you ever used) Emergency contraception? Some examples of names for this are: “Plan B,” “Preven,” “Ella,” “Next Choice,” or "Morning After” pills?*[10]
